# Progress in Medicine

**Published:** 1897-05-01

**Authors:** 


### Progress in Medicine.

### NEUROLOGY.

(*Concluded from p.* 62.)

**Aphasia.**—Bastian[12] has recorded a case of loss of speech in a man, which had existed in a practically unaltered form for over eighteen years. The defects were as follows: His voluntary speech was limited to a very few words, though he could repeat words he heard, and understood everything that was said to him. He spent much of his time reading, and undoubtedly understood what he read. He could not, however, read a single word aloud, nor could he write a single word from dictation, though he could copy the same word readily with his left hand when he had it written before him. There was also some aphemic difficulty of utterance. He subsequently died from a second attack of apoplexy. At the post-mortem examination, atrophy of the convolutions supplied by the left middle cerebral artery was found—involving the supramarginal and angular gyri and the posterior two-thirds of the superior temporal convolution. The whole of this region was occupied by a large pseudo-cyst. Bastian held that the symptoms pointed to an impaired activity of the auditory word centre and defects in the visuo-auditory commisure, and that the post-mortem examination confirmed these views. In discussing this case, Broadbent expressed the opinion that there was no fault in the auditory or visual perceptive centres, for it was only when written or spoken words had to be expressed that failure occurred; and that, the "way out" was not broken, as he could repeat words after anyone. Hence it seemed that both the sensory centres and the motor path were intact, and that the fault was in the path between the sensory and motor centres. It is certainly somewhat difficult to draw any very definite deductions from this case, for extensive compensation must have taken place by the right side of the brain, seeing that there was neither word-blindness nor word-deafness, and yet the angular and supramarginal gyri and the superior temporal gyri were totally destroyed on the left side of the brain. But, admitting this, Broadbent's explanation seems much more probable than Bastian's.

Elder[13] discusses the question of the existence of a special graphic centre in the cerebrum. He admits that no cases could be found in medical literature which clearly proved the existence of a special graphic centre, or the localisation of it, though many cases have been studied clinically tending to show the existence of such a centre. He was of opinion that although the outlines of the letters might at first be traced by the right hand, guided by the visual word centre, in time certain cells became specialised in the neighbourhood of the hand and arm centres for the guiding and co-ordination of these movements. The action of this centre thus determined the character of the handwriting and explained the marked difference between writing with the right and left hands. In all probability the graphic centre only contained the graphic memories of letters and some familiar words, and the letters were combined into speech by the psycho-motor speech centre and the auditory centre, and this was the explanation of the silent articulation which went on when writing. He suggested that possibly this graphic centre, which was really a graphic letter centre, was so small and so near the ordinary hand centre that few lesions could destroy it which would leave the latter intact. He also suggested that those cases of motor aphasia with agraphia where the motor aphasia disappears and the agraphia persists were due, not to a destruction of the graphic centre, but to an interruption in the fibres between Broca's centre and the graphic centre.

Byrom Bramwell, in discussing this paper, said he was inclined to doubt the existence of a special speech centre for writing as distinct from the ordinary psycho-motor centre for the movements of the hands. He agreed with Elder that there was no case on record in which destruction of the second frontal convolution or any other part of the cortex had produced agraphia, and agraphia only. He believed that a localised lesion of the conducting fibres which passed from the motor speech centre to the ordinary motor-centre for the hand and arm would produce an agraphia, but in all cases of complete agraphia known there was, in addition, motor aphasia, or word-blindness, or both, and it was clearly established that destruction of the visual speech centre produces not only word-blindness but also complete agraphia. The whole question turned on the point, could a person who was completely word-blind from destruction of the visual speech centre ever write, *i.e.*, express himself intelligently in written speech by the aid of memories of the kinæsthetic impressions concerned in the act of writing? No such case has been recorded, and until and unless it was, no special graphic centre could be assumed. The memories of kinæsthetic impressions concerned in the act of writing did not seem to be sufficient, in a person who had become word-blind, to enable him to express himself in writing.

Grasset,[14] of Montpellier, has described a unique case under the title, "Aphasia of the Right Hand in a Deaf Mute." He first remarks that Charcot described agraphia as an aphasia of the hand; but his, though apt, is not quite precise. True aphasia of the hand can only occur in the case of a deaf-mute who, never having learned oral speech, loses the faculty of conversing manually. He must not be able to speak orally, and there must not be brachial paralysis sufficient to account *per se* for not speaking manually. These conditions were fulfilled in a case under his care. The patient, who is a congenital deaf-mute, was able to converse manually, using his right hand almost exclusively. Owing to cerebral softening, the result of arterio-sclerosis, he lost the use of his right hand for conversing, though he could use it for the purposes of eating and drinking, grasping objects, &c. The movements of the limb were only partially affected, but the patient's power of expressing himself thereby was gone completely. He could converse with his left hand, and there was no sensory aphasia.

Hinshelwood[15] has recorded a peculiar variety of word-blindness or dyslexia. The patient, a tailor by occupation, aged 45 years, was obliged to give up his work because he became stupid, as he expressed it, when he attempted to do anything. On careful examination it was found that there was a peculiar difficulty in reading, inability to follow his employment, and loss of memory for places. The patient could only read a few words and then came to an abrupt stop, saying he could not go on; if allowed to rest a little he would again read a few words and then stop as before. This was not due to asthenopia, for there was no error of refraction, no affection of the fundus oculi, nor any other ocular defect. And there was no pain or discomfort in the eyes, but only a sense of great mental effort. And on being questioned the patient said he could still see the words distinctly, but could not interpret them The difficulty in his employment was of a nature similar to that experienced in reading. He seemed to have forgotten the relative shapes and positions of the different parts of a garment, and sewed like one who had never learned. And he could not find his way about streets and places previously quite familiar to him. These defects were in all probability dependent on failure of visual memory, though the perceptive faculties remained intact. The case is termed one of dyslexia, after its most prominent feature. This is a term first used by Professor Berlin to denote this special form of word blindness, which has been observed as an early symptom of organic disease of the brain, and also in chronic alcoholics. The patient described by Hinshelwood was probably one of the latter class, for there was a history of alcoholism, and steady improvement and finally recovery took place under total abstinence.

**Hysterical Mutism.**—Michell Clarke[16] gives an account of a case of hysterical mutism of sudden onset, in which the diagnosis was complicated by the presence of tonic spasm of the masseters and abdominal muscles and clonic contractions of the facial muscles. There was in addition concentric contraction of the fields of vision, partial anæsthesia of the back of both arms, and hyperæsthesia of the right side of the chest and the right side of the face and upper part of the neck. The muscular spasm of the muscles of the throat rendered swallowing painful and difficult. The patient recovered in the course of a fortnight, the mutism was succeeded by aphonia, and this by perfect use of the voice. The total loss of articulation in hysterical mutism distinguishes this condition from that of deaf-mutism, in which various noises can be made, and from that of simulatio, in which sounds can be uttered. It is differentiated from all forms of sensory aphasia by the ability to comprehend fully spoken language and to write from dictation, to read written or printed characters and to copy such characters, and to recognise the nature and uses of objects presented to them, and to give correctly in writing the names and uses of such objects. And it is to be distinguished from motor aphasia by the fact that the patient is absolutely dumb; whereas, in motor aphasia. a few words persist, though these may be confined to the utterance of "yes" and "no," and be wrongly employed. And also, in motor aphasia, the power of reading is in most cases lost, and the loss of writing is even more marked than that of speaking. Although in the majority of cases of hysterical mutism the power of writing is preserved, and there is no right hemiplegia, yet in exceptional cases one, or both, of these defects exists; diagnosis is then much more difficult, but it is found that the hemiplegia is of the hysterical type, and that hemianæsthesia also exists—this, taken in association with the fact that the patient is absolutely dumb, enables a correct diagnosis to be made.

**Epilepsy.**—Various results have been reported in reference to Flechsig's treatment of epilepsy by bromides and opium. This consists in giving opium, beginning with five-sixths of a grain daily, distributed in three doses, and steadily increasing this until four or five grains daily are administered. At the end of six weeks the opium is stopped, and bromides begun—in large doses, 75-100 grains a day, for a period of at least two months. The fits generally yield to the first dose of bromide. The treatment should only be adopted in chronic intractable cases of epilepsy in which the administration of bromides alone has failed. The contra-indications are the status epilepticus, plethora, and cerebral focal lesions. This treatment has failed in the hands of some physicians. Thus Pierce Clark[17] comes to the conclusion that Flechsig's treatment is of little value in chronic cases of epilepsy in which dementia has taken place to any marked extent. Flechsig[18] himself has recently reviewed the unfavourable results which have been obtained. He considers that in the fatal cases recorded no causal relation to the treatment has been shown, and points out that a fatal result is common enough in status epilepticus without any opium treatment. His own experience has been fairly satisfactory; thus, six cases out of fifty treated have had no recurrence for two and a quarter years. He points out that if opium be used, the patient must be treated as one who is seriously ill, and that skilled nursing and the most careful medical attendance are essential.

The only other new plan of treatment of epilepsy is by thyroid gland. As regards this, Pierce Clark[19] comes to the conclusion that no benefits result, even though the drug be pushed until toxic effects are produced.

Harris[20] gives an account of two cases of Jacksonian epilepsy, in which the phenomena observed tend to support certain conclusions of Beevor and Horsley derived from experimental stimulation of the monkey's brain. The first case was that of a girl aged five years, who had symptoms of multiple tuberculous tumours in the left rolandic area and in the cerebellum, and who was quite blind from post-neuritic atrophy. In several convulsions observed, there was conjugate movement of the head and eyes to the right, with clonic contractions of the left sternomastoid of the right great pectoral muscle, and of the abdominal muscles on the right side of the body. Hence it would appear that there is a unilateral representation in the cortex even of the abdominal muscles (previously determined by Horsley and Beevor); and that the sternomastoid muscle is an exception to the general rule that the cortex represents movements of the opposite side of the body. The latter phenomenon is readily explained by the fact that the left sternomastoid is concerned in turning the head to the right, and is exactly parallel to what occurs in the case of the eyes; the left side of the cortex innervates not only the right external rectus, but also the left internal rectus, so that the movement of both eyes to the right is brought about by impulses proceeding from the left side of the brain.

The second patient had, in addition to other lesions, thickened and adherent membranes over the lower ascending frontal and parietal convolutions on the left side of the cortex. As a result of this the patient suffered from localised convulsions in the right hand, right side of the face, right side of the tongue, and right side of the soft palate. This agrees with the experimental results of Horsley and Beevor, who found that in the monkey each side of the soft palate had a unilateral representation in the opposite side of the cortex.

**Apoplexy.**—The diagnosis of the cause of apoplexy is sometimes very difficult, and in obscure cases some aid may be obtained from the discovery of Dana[21] that in cases of cerebral hæmorrhage resulting in hemiplegia, the temperature on the paralysed side is higher than on the sound side, whilst in acute cerebral softening from thrombosis or embolism this difference is not present. He has recently recorded some fresh instances which on the whole confirm his generalisation, but admits that very occasionally exceptions occur.

**Insanity.**—Hutchison[22] has recently examined the cortical cells of the brain of a number of individuals who had never suffered from any form of insanity and had died from various diseases. He was led to do this by the fact that pathologists are perhaps rather apt to compare the changes found in the brains of the insane with ideal rather than with actual nerve cells. Pigmentary degeneration was found in more than one-half of the cases examined, but changes in the neuroglia were very infrequent, and marked hypertrophy of the spider cells were never found, and in no cases were any changes found similar to those which occur in general paralysis. Speaking generally, the changes found in the brain cells of the sane are the same in kind as those in the cells of the insane, though widely different in degree.

[12] Bastian. Lancet, Nov. 29, 1896. [13] Elder, Brit. Med. Jour., Jan. 30, 1897. [14] Grasset, Le Prog. Médical, Oct. 31, 1896. [15] Hinshelwood, Lancet, Nov. 21, 1896. [16] Michell Clarke, Clin. Jour., March 10, 1897. [17] Pierce Clark, Amer. Med. and Surg. Bulletin, July 25, 1896. [18] Flechsig, Neur. Centralblatt, Jan., 1897. [19] Pierce Clark, New York Med. Record, Oct. 24, 1896. [20] Harris, Lancet, Oct. 24, 1896. [21] Dana, Post Graduate, New York, vol. xi., No. 7. [22] Hutchison, Edinburgh Hosp. Reports, 1896.

